# From Alarms to Probabilities: Stratified Human Review, Label-Noise Correction, and Calibrated Risk Grading for Industrial Vibration Anomaly Detection

**DOI:** 10.3390/s26175564

**Published:** 2026-09-02

**Authors:** Tao Feng, Kun Chen, Jing Wang, Haonan Guo, Jiewen Wen, Tong Ji

**Affiliations:** School of Computer and Artificial Intelligence, Beijing Technology and Business University, Beijing 102488, China; fengt@btbu.edu.cn (T.F.); 2431062206@st.btbu.edu.cn (K.C.); 2431062194@st.btbu.edu.cn (H.G.); 2431062217@st.btbu.edu.cn (J.W.); 2306030408@st.btbu.edu.cn (T.J.)

**Keywords:** risk grading, human-in-the-loop, label noise, Rogan–Gladen estimator, alarm management, anomaly detection, condition monitoring

## Abstract

Industrial anomaly detectors emit binary alarms, but production lines need graded dispositions. Calibrating alarms into fault probabilities requires ground truth, coming only from noisy human review treated as exact. We report a deployed closed loop on a reciprocating-compressor line (46,023 units, nine test campaigns, four fused detector legs). A stratified review of 1161 units audited a fusion-score grading and refuted its assumed monotonicity: precision was 25.2%/13.8%/26.1% for high/medium/low tiers. The cause was correlated false positives: two legs firing on shared broadband transients agreed on 480 units at 21.0% precision—detector agreement is not independent evidence—whereas one periodicity feature was monotone (27.8% → 60.0% → 100%). A blind test against seeded fault units (hardware ground truth) measured reviewer sensitivity at 0.905 and specificity at 0.421 on hard cases; Rogan–Gladen correction restored monotonicity (95% of bootstrap replicates; 83% under campaign-cluster resampling), exposed the low-tier advantage as a label-noise artifact, and re-estimated no-alarm prevalence at 4–10% versus the observed 13.3%. Corrected evidence drove a redeployed rule calibrating tiers at ≈67%/27%/17% under review budgets (≤1%/≤3%/≤9% of production). The methodology—stratified audit, seeded-fault blind testing, and evaluation-side prevalence correction—transfers to any human-verified system.

## 1. Introduction

As core components in automotive supply chains, energy development, and industrial manufacturing, the energy efficiency and operational reliability of refrigeration and air-conditioning compressors play a decisive role in the quality of end products and the overall performance of the system [1,2,3]. Therefore, end-of-line (EOL) vibration screening is typically conducted to reject defective units [4,5].

In recent years, machine learning-based anomaly detection methods have been widely applied in industrial quality control [6,7,8]. These methods typically score each unit based on a single measurement and classify it as normal or anomalous [9]. However, decision-making requirements in actual production environments are often more complex than binary classification: managers need to adopt different disposition measures according to risk levels, such as immediate removal from the line, same-shift review, or sampling inspection [10]. In this context, how to transform detector alarms into risk grading with probabilistic significance has become a critical yet rarely studied problem.

End-of-line acceptance testing for mass-produced mechanical products increasingly relies on learned anomaly detectors: each unit is measured only once, scored, and then flagged as anomalous or passed [10,11]. However, the output of the detector is a binary alarm, whereas the decision that the production line must make is graded: immediately pulling the unit off the line, arranging for the shift inspector to re-listen on the same day, or merely logging it for subsequent sampling inspection. Between the alarm and the disposition lies a rarely measured quantity: the probability that an alarmed unit is actually faulty. When alarms from multiple detectors are fused [12], engineering practice commonly defaults to two assumptions: (i) the more detectors that alarm simultaneously, the higher the fault probability; and (ii) the human re-inspection used for verification is reliable enough to serve as ground truth [13]. These two assumptions lack empirical validation in practical applications. This paper reports a deployed industrial case in which both of the aforementioned assumptions are measurably falsified, and proposes an audit-and-correction methodology to replace them.

This study focuses on a vibration screening system for a reciprocating refrigerator compressor production line, which fuses four heterogeneous detector legs (an impact classifier, a spectrogram CNN, a periodic-noise feature, and a high-frequency burr feature). Through data analysis of 46,023 units, we found that the first-generation risk grading rule (based on multi-detector weighted scoring) failed to achieve the expected risk monotonicity. To investigate the reasons, we designed a stratified human review scheme involving 1161 units and introduced a hardware-level ground truth blind test to quantify the reviewer’s operating characteristics [14]. Furthermore, we applied the Rogan–Gladen correction method to adjust for label noise and rewrote the grading rule based on the corrected evidence to meet the review capacity constraints of the production line.

The main findings of this study include the following: (1) Multi-detector agreement exhibits anti-informativeness under shared confounders—the precision of simultaneous triggering by the impact classifier and the spectrogram CNN (21.0%) is lower than that of the single periodicity feature (40.0%), revealing the negative impact of correlated errors on fused decisions; (2) Human review labels contain significant noise (sensitivity 0.905, specificity 0.421), and after Rogan–Gladen correction, the monotonicity of risk grading is restored; (3) The grading rule rewritten based on corrected evidence and capacity constraints achieved risk grading of ≈67%/≈27%/≈17%, and the closed-loop deployment was completed within days.

The remainder of this paper is organized as follows: Section 2 reviews the current research status in related fields; Section 3 introduces the system background and problem setting; Section 4 elaborates on the stratified review design, blind test methodology, and Rogan–Gladen correction method; Section 5 presents the main research results and discusses the deployment and closed-loop application of the corrected rules; Section 6 discusses the limitations, scope of application, and transferability of the study; and Section 7 concludes the paper.

## 2. Related Work

**Alarm Management and Rationalization.** Process-industry alarm management standards [15,16] address alarm flooding and prioritization through design-stage consequence/urgency matrices—ordinal categories assigned during rationalization, rather than measured probabilities. Academic research focuses on quantifying alarm system performance (alarm rates, chattering, flood analysis) or deriving false/missed-alarm rates and delays model-based at design time to tune deadbands and delay timers [17,18]. Recent work structures rationalization with causal/graph models and notes the absence of data-driven priority updating mechanisms [19]. Our tier probabilities are instead measured post-deployment by stratified review of the running system, and our capacity budgets (1%/3%/9% of production) play the role that alarm-rate targets play in those standards—but each budgeted tier carries an audited, corrected fault probability. Bayesian-network approaches derive fault-management priorities from inference over a process model whose parameters are learned from operational data [20]; the tier probabilities reported here are instead measured directly by stratified review of the running system and corrected for measured reviewer error.

**Review Budgets and Human-in-the-Loop Anomaly Detection.** The idea of an explicit human-verification budget appears in security analytics: AI$\hat{2}$ processes a daily “investigative budget” of top-ranked events, whose analyst labels retrain the detector [21]. Active anomaly discovery optimizes which instances to show an expert under a labeling budget [22]. These budgets are training-time labeling budgets: human effort is spent to improve the detector, labels are taken at face value, and only top-ranked (alarmed) items are ever reviewed. Our budget is a run-time disposition budget: the detector is frozen, human effort is the resource allocated by the grading layer itself, review outcomes are treated as noisy measurements, and—critically—the un-alarmed stream is also audited (the control stratum). None of the aforementioned works achieve this, and without it, the missed-detection side cannot be priced. Annotator-quality models in the crowdsourcing tradition estimate confusion matrices without ground truth via redundant labeling [23]; we deliberately bypass that inference by directly measuring the reviewer against physical ground truth.

**Prevalence Estimation, Quantification Learning, and Noisy Evaluation.** The learning-with-noisy-labels literature concentrates on training robustness [24,25]. On the evaluation side, the Rogan–Gladen estimator [26] is the classical correction of an observed positive rate for imperfect test sensitivity/specificity; confidence procedures for the corrected prevalence exist [27], including degeneracy conditions at extreme prevalence that our low-rate pools approach (we handle them by truncation and report the sensitivity explicitly, Section 5.6). The same algebra reappears in quantification learning as the adjusted-classify-and-count rule for estimating class prevalence from an imperfect classifier [28]. We therefore claim no novelty for the estimator itself. What we found no precedent for is the measurement design around it: the “imperfect test” is a human reviewer, whose operating characteristics are measured by a blind test keyed to physically seeded faults, and the corrected quantities are the disposition-tier probabilities of a deployed detection system, corrected pool-by-pool because reviewer difficulty—and thus *f*—varies across pools. Where ground truth is genuinely absent, latent-class methods estimate test accuracy jointly [29,30]; seeding hardware faults converts that inference problem into a direct measurement, at the cost of maintaining the seeded units—an asset test that lines often already hold for gauge repeatability studies [31].

**Seeded-Defect Blind Testing.** The probability-of-detection (POD) tradition in non-destructive testing evaluates human inspectors on specimen sets with implanted defects of known size [32], and measurement-systems analysis audits attribute gauges (including human judges) against reference parts [31]. Our blind test is this tradition’s instrument; the step we have not found elsewhere is coupling the measured (*a*, *b*) to a prevalence correction of a machine-learning system’s alarm pools.

**Correlated Errors and Common-Cause Failure.** That nominally redundant channels fail dependently is foundational in reliability engineering (common-cause failure models such as the *β*-factor approach [33]), and this was established for computational redundancy by multiversion-programming experiments: independently developed programs fail on the same “hard” inputs far more often than independence predicts [34,35,36]. In ensemble learning, error correlation is known to bound fusion gains [37]; distributed-detection theory designs optimal fusion rules given dependent local decisions [38,39]. The analogy itself is thus not new; our contribution is a quantified field instance in condition monitoring—agreement-pool precision (21.0%, *n* = 386) below the better single leg (40.0%), with the shared confounder physically identified (broadband transients and corrupted recordings)—together with its operational consequence: voting logic inherited from hardware-redundancy practice cannot be presumed safe for fused learned detectors. We found no condition-monitoring publication reporting such an inversion in a production system; fusion papers in this space uniformly report false-alarm reduction. A recent research note argues that deployment decisions should report the absolute number or hourly/daily rate of false alarms that a detector produces, not only accuracy-family metrics [40]; that argument concerns the false-alarm burden of detectors considered individually, and it does not address whether the false alarms of different detectors are statistically dependent—the question that our agreement-pool analysis answers.

**Industrial Acoustic Anomaly Detection.** The DCASE line established datasets and benchmarks for machine-sound anomaly detection [41]; evaluation is detection-accuracy-centric (AUC on labeled test sets). To the best of our knowledge, disposition-grade calibration, review-capacity budgets, and reviewer-noise correction are not addressed in this line. Our group has previously addressed cross-condition transfer for scroll-compressor anomaly detection at the model level [4]; the present work concerns a different machine family and a different layer of the problem—the decision logic built on top of an already deployed fused detector, and the reliability of the human review used to calibrate it.

## 3. System Context and Problem Setting

### 3.1. Production Line and Detection System

The line performs end-of-line vibration screening of reciprocating refrigerator compressors (anonymized manufacturer). Each unit is measured once on a test fixture equipped with a single-axis accelerometer (*f_s_* = 51.2 kHz); the first 2.0 s of the record are analyzed. The present work and our group’s published end-of-line screening platform [4] belong to the same research programme but concern different compressor families and operating conditions (automotive scroll compressors tested at 1000 and 3000 r/min in [4]); only the audit-and-calibration methodology is shared between the two. This study covers 46,023 units from nine test campaigns (anonymized campaign IDs 3D28, C12D, C72C, C7CE, 7C0B, 99F1, 802D, 55C7, and 2DBF) collected over ten production days (14–23 June 2026). A campaign is a test task opened on the line at a given time; all nine ran on the same production line, are separated in time, and may differ in the product model under test. Campaign is therefore the natural grouping unit in this study—it bundles the product model, the fixture state, and the time window—and it is the level at which per-group results are reported throughout. No production labels are used; the system runs purely on model predictions. Product-model composition varies across campaigns (anonymized as Families A/B; Supplementary Table S12); the campaign-cluster bootstrap (Section 5.6) and the per-campaign precision probe (Supplementary Table S6) show that the tier-ordering and demotion conclusions are stable across this variation, with the low-versus-baseline link as the only weak element.

Each unit is tested once on a pallet-located end-of-line station. Housing vibration is picked up by a single-axis charge-mode piezoelectric accelerometer (SAPD0500, charge sensitivity 15 pC/g, 0.5–10 kHz)—a charge-output sensor that requires an external charge amplifier (not IEPE/ICP)—pressed against the top of the compressor shell by a pneumatic carrier, and conditioned by one channel of a three-channel charge amplifier (SAPE04-B, gain 10 mV/pC, low-pass 10 kHz), giving an overall chain sensitivity of 150 mV/g (full scale ≈±33 g at the ±5 V analogue input, chosen to accommodate carrier-contact and fixture transients; compressor body vibration is 0.1–0.5 g RMS). The conditioned voltage is digitized by one channel of a four-channel NI 9234 data-acquisition module (24-bit delta-sigma ADC, 51.2 kS/s per channel, ±5 V input range, IEPE excitation disabled) at $f_{s}=51.2$ kHz; a LabVIEW acquisition program records 3.0 s per unit (153,600 samples) as 32-bit floating-point waveforms; all four detector legs in the study version analyse the first 2.0 s (102,400 samples). Pre-processing is per leg: mean removal for all legs; for the spectrogram CNN, an STFT (1024-point Hann window, 50% overlap) in two bands (64–4096 and 4096–12,800 Hz), log-energy, per-band clipping at the 99.5th percentile and z-scoring; for the impact classifier, band-pass filtering followed by Hilbert-envelope features. The 4096–12,800 Hz band of the spectrogram front end therefore extends beyond the analogue passband; content above the amplifier cut-off is attenuated rather than absent, and the periodicity (phf) fault band on which the monotone leg operates lies inside the passband. The band edges are properties of the STFT front end as deployed, not of the analogue chain. No whole-signal filtering or amplitude calibration is applied. The detection software is a Python inference service (version v15w2clip_ds3v3, trained 30 June 2026); the grading rule studied here is its post-processing module (v2, 3 July 2026).

Four detector legs are computed per unit and fused by disjunction (Table 1). Leg internals are companion-paper subjects; this paper treats each leg as a black-box trigger and studies the decision layer built on the fused alarm stream. The four legs are held fixed throughout: the v1-versus-v2 comparison in Section 6.2 is a like-for-like ablation on identical units and identical labels, with no detector, threshold, or datum changed. The conclusions of this paper therefore do not depend on the internal implementation of any leg.

An alarm is raised if collision, ds3v3, or phf triggers. The burr leg does not alarm; burr-only units enter an offline re-check queue. Over the study window the system alarmed on 4087 units (8.9%) and queued 485 more (1.05%), for a non-normal total of 4572 (9.9%).

### 3.2. The Grading Requirement

A 9%-alarm stream is not actionable per unit. The line’s review capacity, not the detector, is the binding constraint. Operations therefore specified capacity budgets from the available review staffing and shift-time capacity (how many units per day can be immediately inspected or re-listened), not from detector performance: at most 1% of production may demand immediate action, at most 3% cumulative may require same-shift review, and the total alarm stream must stay within 9%. These budgets have an internal-consistency rationale: the excess of the alarm rate over the false-alarm floor estimated on a known-clean campaign (8.9%–∼6.1% ≈ 2.8%) approximately matches the high+medium budget (1% + 2%). Each tier maps to a disposition (Table 2).

The scientific question is then as follows: what fault probability does each tier actually carry? Answering this requires human review, and trusting that answer requires knowing how good the review itself is. Section 4 and Section 5 address both.

### 3.3. First-Generation Grading (v1): The Audited Baseline

v1 followed the standard fusion intuition. Each triggered leg contributes an evidence score: one point at threshold, growing to two with strength; the CNN’s four classes map to 0/0.5/1/2; burr contributes half weight. The total score *R* is cut at population quantiles calibrated so that the high tier is 1% of production and high+medium is 3%. Concretely, R≥3.03 defines the high tier (461 units), R≥1.75 defines the medium tier (919 units), remaining alarms form the low tier (2707 units), and burr-only units form the sampling tier (485 units). By construction, the v1 high tier is dominated by multi-leg agreement: 93% of it consists of collision∧ds3v3 co-triggers. The exact per-leg scoring functions and all threshold values (*R* ≥ 3.03/1.75; 0.468; class > normal; phf > 30; burr > 6.3) are reproduced verbatim in Supplementary Code S13 with pseudocode, making the grading fully reproducible.

## 4. Methods

**Figure 1:**
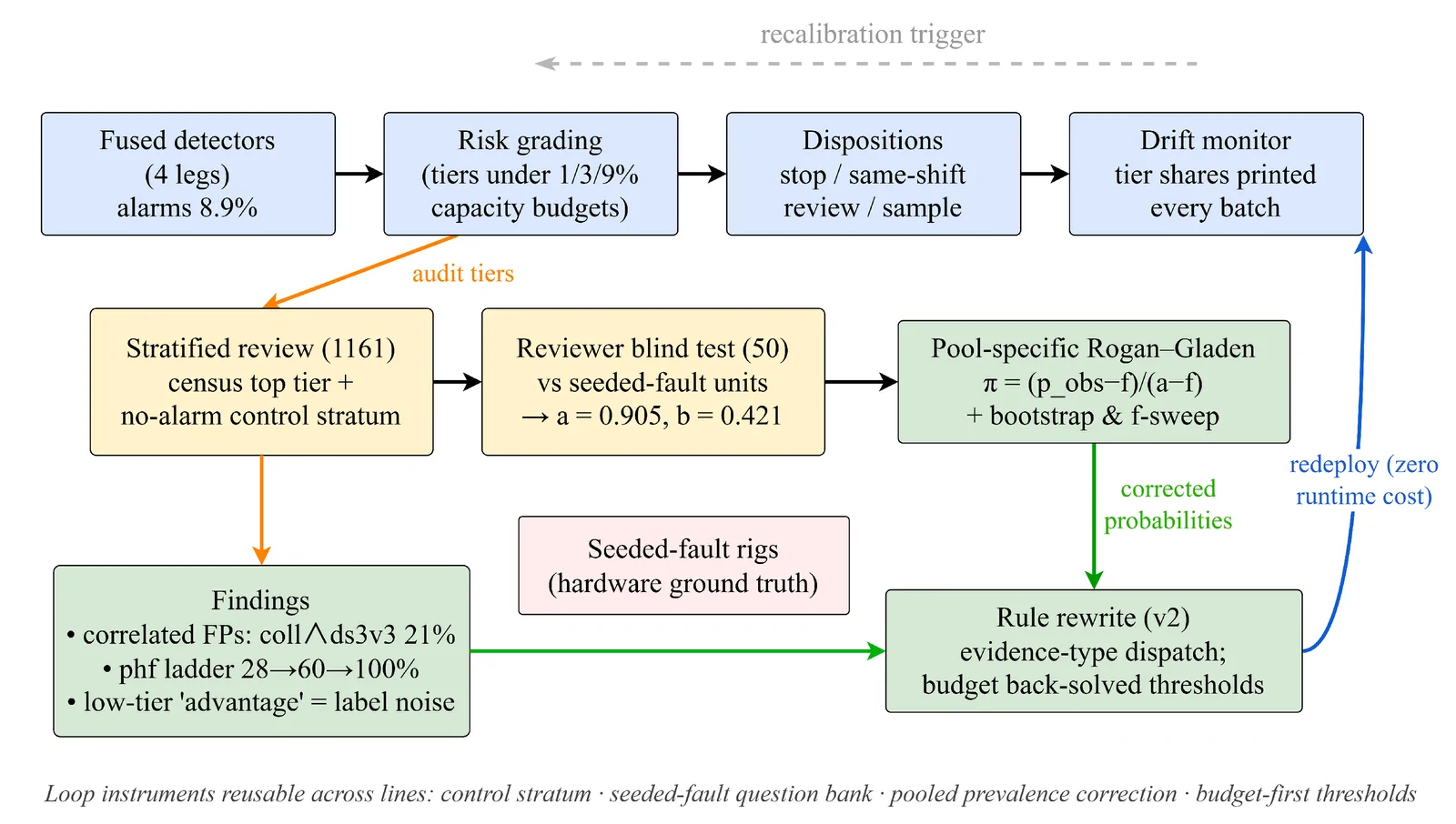
The audit–measure–correct–redeploy loop. Production flow (top row): four fused detector legs alarm on 8.9% of units; a grading layer assigns risk tiers under review-capacity budgets (high ≤1%, high+medium ≤3%, total ≤9% of production), each tier mapped to a disposition; per-batch tier shares serve as a drift monitor. Calibration flow (middle and bottom): a five-stratum review of 1161 units (census of the top tier plus a no-alarm control stratum) audits the tiers; a 50-recording blind test against seeded-fault units (hardware ground truth) measures reviewer sensitivity a=0.905 and specificity b=0.421; pool-specific Rogan–Gladen correction with bootstrap and *f*-sweep uncertainty propagation converts observed precisions into corrected prevalences; the resulting findings (correlated false positives, the phf evidence ladder, the label-noise origin of the apparent low-tier advantage) drive an evidence-type rule rewrite whose thresholds are back-solved from the budgets, redeployed at zero runtime cost. Instruments in the loop are line-agnostic and reusable.

The overall methodology, structured as an audit–measure–correct–redeploy loop, is illustrated in Figure 1. This framework connects the production flow (where fused detectors generate alarms and grading layers assign risk tiers under capacity budgets) with the calibration flow (where stratified review and blind testing measure and correct label noise to rewrite the grading rule).

### 4.1. Stratified Review Design

We drew a five-stratum review sample of 1161 units (fixed seed, reproducible; stratum sizes chosen to fit one review shift; Table 3; Supplementary Table S1). Stratum sizes were fixed before review to give usable precision: for *n* = 200 the Wilson half-width is ≈±6.0 points at an anticipated precision of 25% (±4.7 points at the 13.3% control rate), sufficient to resolve the observed tier contrasts; the high tier was censused (461/461). Medium samples were stratified by trigger pattern and low samples by trigger leg × campaign (fixed seed), so every dominant pool obtained direct review coverage (Supplementary Table S11).

The control stratum is essential and often omitted in practice: it prices the missed-detection rate (the fault prevalence among units the system passed) and anchors the baseline against which tier enrichment is judged.

A trained line reviewer audited each unit with a purpose-built review workbench (audio playback with synchronized spectrogram display; single-keystroke label entry; per-stratum work folders exported to and re-imported from CSV, so the annotation round-trip is lossless), assigning one of the following: normal; a fault category (impact/collision, friction, warble, periodic, squeal, other); invalid recording; or undecidable. The operational positive class is actionable non-conformity, defined as any label requiring operator action (fault categories and invalid recordings, the latter requiring re-test). Undecidable units (n=94 overall: 53/19/12/5/5 across strata (i)–(v)) are excluded from precision denominators. We report Wilson 95% intervals throughout [46].

Population-level tier probabilities use stratum weights $w_{h}=N_{h}/n_{h}$ (Horvitz–Thompson): w=1 (census), 4.59 (stratum ii), 13.54 (stratum iii), 4.85 (stratum iv). Interval estimates for the weighted quantities come from the stratified bootstrap of Section 5.6; unweighted Wilson intervals are shown alongside for transparency.

### 4.2. Measuring the Reviewer: Blind Test Against Seeded-Fault Ground Truth

Review labels are the only ground truth available at scale, but they are fallible precisely on the borderline cases that dominate alarm pools. To measure this, we constructed a 50-recording blind test (Supplementary Table S2) from campaign 99F1’s collision-triggered stream, deliberately chosen as the hardest slice (borderline impact signatures; collision-leg probabilities 0.217–0.978; loudness-matched so that level cues are uninformative). Ground truth is hardware-level: 25 recordings come from units into which faults had been deliberately seeded (fault-seeded rigs), and 25 from true-normal units; the answer key derives from the physical state of the unit, not from any annotator. The reviewer received shuffled, unlabeled files with instructions to judge each independently (collision/normal/undecidable, plus a confidence rating and free-text note) and to prefer “undecidable” over guessing.

Outcomes (recomputed from the raw answer sheet and key): of 25 true-fault recordings, 19 were judged non-normal, 2 normal, and 4 undecidable; of 25 true-normal, 8 normal, 11 non-normal, and 6 undecidable. Excluding Undecidables: sensitivity a=19/21=0.905 (Wilson 95% CI 0.711–0.973), specificity b=8/19=0.421 (Wilson 95% CI 0.231–0.637; Clopper–Pearson intervals in Supplementary Table S10), binary accuracy 27/40=68%. Treating all undecidable responses as errors moves *a* to 0.76 and *b* to 0.32; across the three undecidable mappings the control-stratum corrected interval is 4.1–9.7% (baseline), 5.1–11.7% (all-wrong) or 4.1–9.6% (all-correct), so the 4–10% conclusion is insensitive to the mapping choice (Supplementary Table S10); propagating *b*’s posterior (Beta(8.5,11.5), f=1−b upper arm) widens the corrected intervals but leaves every directional conclusion unchanged; under the pure hard-case extreme f=1−b the collision∧ds3v3 pool’s corrected value is pinned at 0, so the “agreement pool below medium” conclusion is only reinforced. (Answers of “abnormal noise” or “invalid” on true-fault items count as non-normal; the mapping is fixed in the analysis script.) Self-rated confidence was informative (high-confidence judgments, n=36: 72% accuracy; medium, n=4: 25%; recomputed from the raw answer sheet), suggesting confidence-weighted re-review by the same reviewer as a refinement.

Two scope statements follow from the design. First, a=0.905 is plausibly global: Even on the hardest slice the reviewer missed only 2/21 true faults, so observed pool precisions are barely deflated by missed faults. Second, b=0.421 is a hard-case bound, not a global rate: applying it globally would predict a ≥58% non-normal rate in any pure-normal pool, which the main review contradicts (medium 13.8%, low 26.1%, control 13.3% observed). The blind-test slice, consisting of loudness-matched borderline impacts, represents the upper end of the mislabel-rate range.

### 4.3. Pool-Specific Rogan–Gladen Correction

Let $p_{\mathrm{obs}}$ be the observed (review-scale) positive rate in a pool, *a* the reviewer sensitivity, and *f* the reviewer false-positive rate in that pool (f=1−specificity on that pool’s difficulty mix). The classical Rogan–Gladen estimator [26] recovers the true prevalence:

(1) $$\pi =\frac{p_{\mathrm{obs}}+b-1}{a+b-1}=\frac{p_{\mathrm{obs}}-f}{a-f},$$

truncated to [0,1]. Identifiability requires a>f (the reviewer beats chance in that pool); $p_{\mathrm{obs}}<f$ yields π=0 after truncation. Because *f* varies with pool difficulty, we correct pool by pool with interval-valued *f*:

**Acoustically Distinct Pools** (phf-dominated tiers, sampling tier, control): f∈[0.05,0.10]. Rationale: these pools are dominated by clearly audible signatures (periodic squeal) or ordinary normals; the main-review internal-consistency argument above bounds *f* well below the blind-test value.

**Hard-Case-Dense Pool** (collision∧ds3v3 agreement, the population the blind-test slice was drawn from): f∈[0.15,0.30]. The blind-test value f=0.579 is the pure hard-case extreme; the deployed pool mixes such borderline impacts with easier members (clear knocks, obvious bad recordings), and the interval corresponds to roughly one-quarter to one-half of the pool being blind-test-hard (0.25–0.5×0.579≈0.14–0.29). The sensitivity curves over the full range f∈[0,0.6] in Section 5.6 make the dependence of every conclusion on this choice explicit. These intervals are anchored by data rather than rationale alone: identifiability requires $f<p_{\mathrm{obs}}$ in each pool (e.g., the control stratum’s $p_{\mathrm{obs}}=13.3\%$ bounds f<0.133); the blind test supplies the hard-case upper end (f=0.579); and the per-pool invalid-recording shares bound the easy-material mislabel rate (Supplementary Table S8). Per-pool sweeps (Supplementary Figure S7) report the *f* at which each conclusion flips: medium to 0.19, low to 0.12 (adopted upper bound 0.10, a thin but positive margin), control “low > baseline” below 0.03; high, sampling and the agreement pool never flip within [0,0.6).

Uncertainty in *a* (19/21) and sampling error in $p_{\mathrm{obs}}$ are propagated by stratified bootstrap (B=10,000; within-stratum resampling; *a* drawn from its Jeffreys posterior Beta(19.5,2.5); *f* drawn uniformly from the pool’s interval; Section 5.6). We emphasize what the correction can and cannot change: under pool-wise independent mislabeling, correction rescales contrasts monotonically within a pool type but can reorder pools only when their *f* differs, which is precisely the effect at issue between impact-heavy and phf-heavy pools.

### 4.4. Rule Synthesis Under Capacity Budgets (v2)

The v2 grading rule was rewritten from the audited evidence (Section 5), replacing score summation by evidence-type dispatch:

- **High:** phf triggered and (phf ≥40 or ds3v3 also triggered).
- **Medium:** Other phf triggers (phf ∈(30,40)); or collision∧ds3v3 agreement with collision probability ≥0.9.
- **Low:** All remaining alarms.
- **Sampling:** Burr-only (unchanged).

Thresholds were chosen on the audited data to preserve the capacity budgets on the 46,023-unit population (high 320 = 0.70%; high+medium 2.74%; total alarms 8.88%). A subsequently added enhancement leg (an open-set spectral outlier score, “mel”; details in Section 6.3) promotes low-tier units to medium only within the remaining budget: its threshold was back-solved so that high+medium landed exactly on 3.00%. This “budget-first” thresholding is deliberate: the grading layer is pure post-processing, so its operating point is set by review capacity, not by detector ROC geometry.

## 5. Results

### 5.1. The v1 Audit: Assumed Monotonicity Refuted

**Figure 2:**
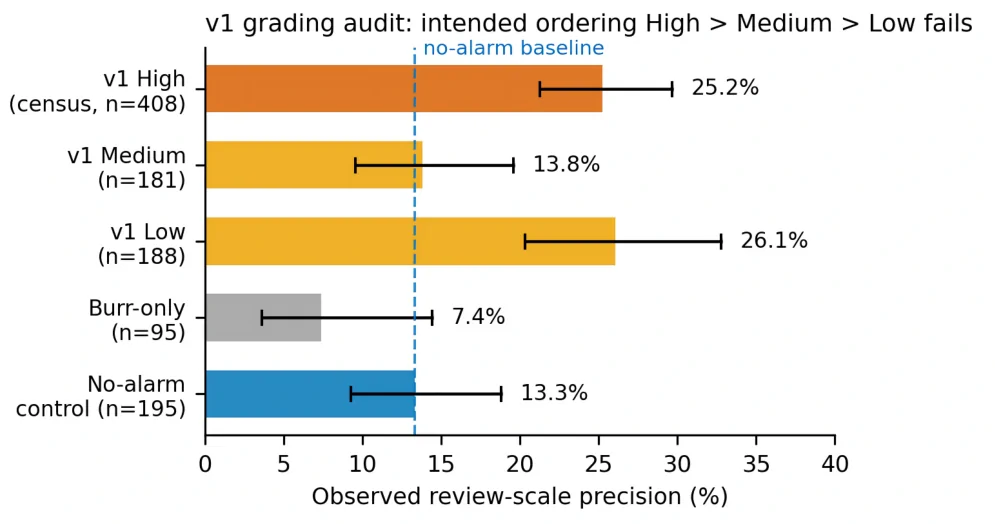
The v1 grading audit refutes the assumed tier ordering. Observed review-scale precision (bars) with Wilson 95% intervals (black whiskers) for the five review strata; the dashed line marks the no-alarm control stratum (13.3%). The v1 high tier (25.2%) barely exceeds the baseline, and the low tier (26.1%) apparently outperforms the medium tier (13.8%)—the intended high > medium > low ordering fails. Undecidable annotations are excluded from denominators (*n* shown per stratum). All values recomputed end-to-end from the raw review annotations. Undecidable exclusions per stratum: 53/19/12/5/5, giving denominators 408/181/188/95/195.

Observed (review-scale) precision by v1 tier, with undecidables excluded, is reported in Table 4 and Figure 2.

The intended ordering high > medium > low fails twice: the high tier barely beats the no-alarm baseline, and the low tier apparently outperforms the medium tier. A grading built on the multi-detector-agreement premise, calibrated only for rates (1%/3%/9%), carried no probability meaning at all. Campaign heterogeneity was extreme: v1 high-tier precision ranged from 82% (14/17, campaign 2DBF) down to 12% (15/126, 7C0B) and 10% (3/29, C12D) (Supplementary Table S3). Because campaigns are separated in time and may carry different product models, this spread bundles product, fixture-state and temporal variation rather than unit-to-unit variation between parallel machines. Notably, campaign 7C0B’s excess of multi-leg agreements, initially suspected to indicate a genuine bad batch, turned out to be an excess of correlated false alarms (Section 5.2).

**Table 5:** v2 tier calibration on the audited data (Horvitz–Thompson reweighting). All three capacity budgets hold and precision is strictly monotone.

v2 Tier	Population Share	Observed (Unweighted)	Calibrated (Review Scale)	Wilson 95% (Unweighted *n*)
High	320 (0.70%)	69.6% (39/56)	66.8%	57–80% (39/56)
Medium	940 (2.04%; cum. 2.74%)	29.8% (50/168)	27.0%	23–37% (50/168)
Low	2827 (6.14%; cum. 8.88%)	15.9% (88/553)	17.2%	13–19% (88/553)
Sampling	485 (1.05%)	7.4% (7/95)	7.4%	4–14% (7/95)
(No-alarm baseline)	—	13.3% (26/195)	13.3%	9–19% (26/195)

### 5.2. Correlated False Positives: Agreement Is Not Independence

**Figure 3:**
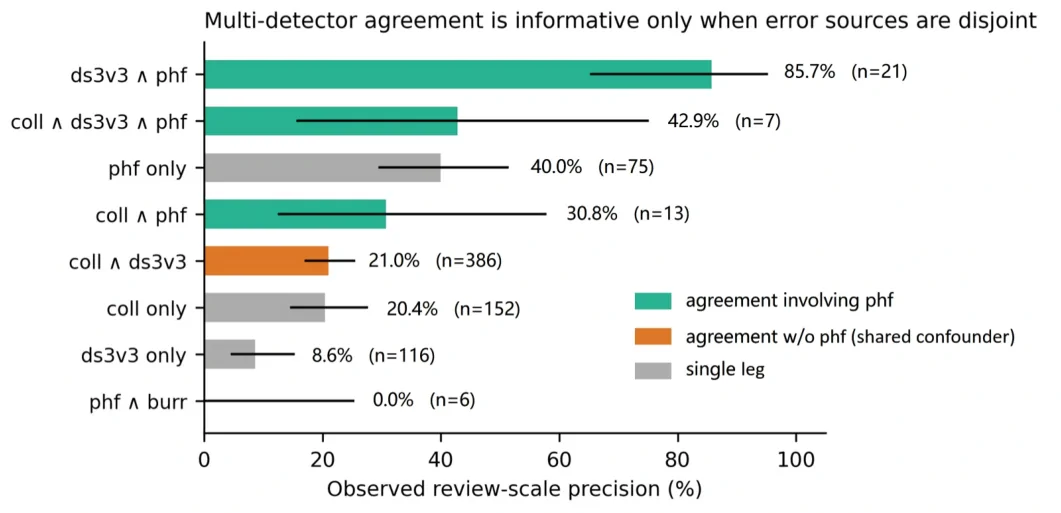
Multi-detecto r agreement is informative only when error sources are disjoint. Observed review-scale precision by trigger pattern (strata (i)–(iii) pooled; Wilson 95% whiskers; *n* per bar). Agreement patterns that include the periodicity feature phf (green) reach 85.7% precision, whereas the collision∧ds3v3 agreement (red, n=386)—the pattern that dominated the v1 high tier—achieves only 21.0%, below the single phf leg alone (40.0%) and indistinguishable from collision alone (20.4%): the two legs respond to the same broadband-transient confounders, so their agreement is correlated error, not independent confirmation.

Decomposing precision by trigger pattern (strata (i)–(iii) pooled, undecidables excluded) yields Table 6 and Figure 3.

The pattern that dominated the v1 high tier—namely collision∧ds3v3—has lower precision (21.0%) than the single phf leg alone (40.0%), and essentially the same as collision alone (20.4%): the CNN’s agreement added nothing. The mechanism is a shared confounder: both legs respond to broadband transient energy, whether from an internal impact, an external knock, fixture rattle, or a corrupted recording. Direct Evidence: Of the 55 reviewed units in the alarmed strata labeled invalid recording, 51 fall in collision-participating patterns (recomputed from the raw annotations; the source engineering report quoted 49). The impact leg was therefore in part acting as a bad-recording detector. Agreement involving phf behaves oppositely (ds3v3∧phf: 85.7%), because phf’s confounder set (periodic squeal) is disjoint from the transient confounders. Multi-detector agreement is informative exactly when the legs’ error sources are disjoint; under shared confounders it is anti-informative relative to the better single leg, constituting the detection-side analogue of common-cause failure in redundant channels.

**Figure 4:**
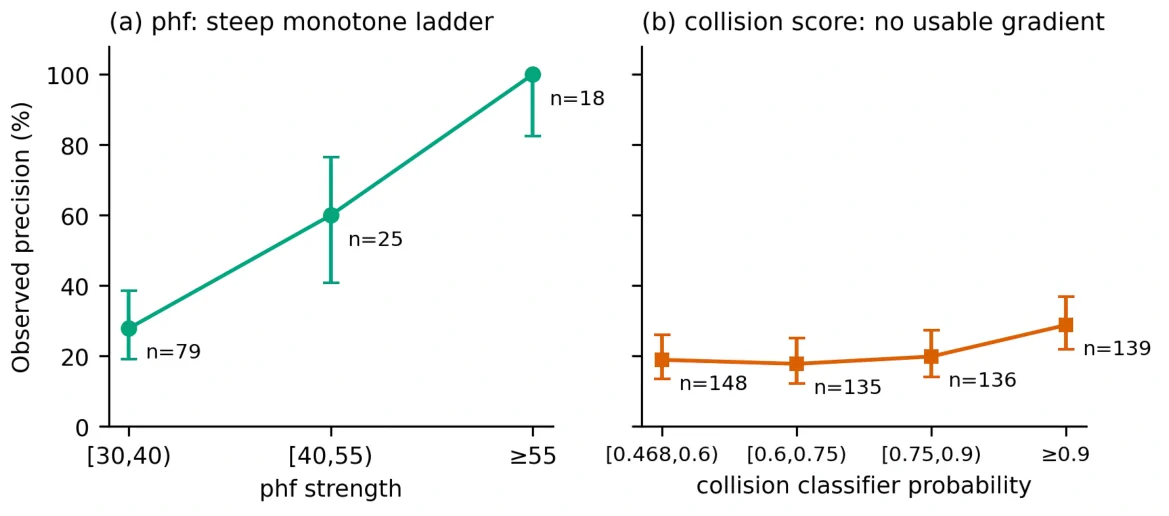
One evidence axis is steeply monotone; the other is flat. (**a**) Observed precision of phf-triggered units by phf strength: 27.8% → 60.0% → 100% (Wilson 95% whiskers; the ≥55 bin is a small census, n=18). (**b**) Observed precision of collision-triggered units by collision-classifier probability: 18.9%, 17.8%, 19.9%, 28.8% across bins from the 0.468 trigger threshold to >0.9—score magnitude carries almost no incremental evidence. This asymmetry motivates the v2 rule: phf strength dominates tier assignment; the collision probability appears only as the ≥0.9 qualifier for medium-tier admission of collision∧ds3v3 agreements.

A second null finding sharpens the picture. Within the collision leg, the classifier’s own probability carries almost no incremental information: precision across four probability bins from 0.47 to >0.9 is 18.9%, 17.8%, 19.9%, and 28.8% (Figure 4b)—a shallow rise in which only the top bin lifts above the pooled 21%. By contrast, phf strength is steeply monotone (Section 5.3). Score magnitude is not evidence quality; the v2 rule accordingly uses the collision probability only once, as the ≥0.9 qualifier on collision∧ds3v3 agreements admitted to the medium tier.

### 5.3. A Single Monotone Evidence Axis: The Phf Ladder

Among phf-triggered reviewed units (strata (i)–(iii), undecidables excluded), precision rises steeply with phf strength (Table 7; Figure 4a).

This is the only steeply monotone strength–precision relationship in the system, and it directly supplies the v2 high/medium boundary (phf ≥40, strengthened by ds3v3 agreement whose error sources are disjoint from phf’s). We report the ≥55 cell honestly as a small-sample census (n=18) with its Wilson interval; the design decision it supports (phf dominates grading) rests on the whole ladder, not on the 100% point.

### 5.4. v2 Calibration on the Audited Data

Applying the v2 rule to the reviewed sample and reweighting by stratum (Horvitz–Thompson) yields Table 5. These are calibration results on the audited development data and should be interpreted as such, not as independent validation; prospective confirmation is provided by the deployed drift monitor (Section 6.1) and, where available, post-deployment review batches.

Observed (unweighted) and Horvitz–Thompson weighted estimates are reported together for every tier (Table 4, Table 5 and Table 8). The audit findings (Section 5.1) are driven by observed stratum precisions; the calibrated tier probabilities (this section) are driven by the weighted estimates. Terminology: *precision* = review-scale positive rate among reviewed units; *corrected prevalence* = population fault rate after Rogan–Gladen correction; *calibrated probability* = the tier probability quoted for dispositions.

All three budgets hold (0.70%≤1%; 2.74%≤3%; 8.88%≤9%) and precision is monotone: 67%>27%>17%>13% baseline. In operator language: high ≈ two of three units truly non-conforming; medium ≈ one in four; low ≈ one in six. Stratified-bootstrap 95% intervals for the weighted estimates (66.8% → 48–83%; 27.0% → 17–37%; 17.2% → 13–22%; baseline 13.3% → 9–18%) are consistent with the unweighted Wilson intervals shown; the high tier’s interval is wide because the v2 high pool contains only 56 reviewed units after re-grading.

Because the same audited data were used for rule development and estimation, we performed leave-one-campaign-out (LOCO) validation: for each of the nine campaigns the v2 thresholds were re-fit on the reviewed units of the remaining eight campaigns and applied to the held-out campaign. In all nine folds the re-fit recovered the deployed thresholds (phf ≥40; collision probability ≥0.9), and the full-data re-fit exactly reproduced the deployed values (40/0.9/mel 0.473), indicating no threshold-selection optimism for the tier thresholds (the budget-back-solved mel promotion threshold varies 0.34–0.70 across folds; Section 6.3) (Supplementary Table S5).

### 5.5. Label-Noise Correction: Monotonicity Is Real, and the Pessimism Was Inflated

**Figure 5:**
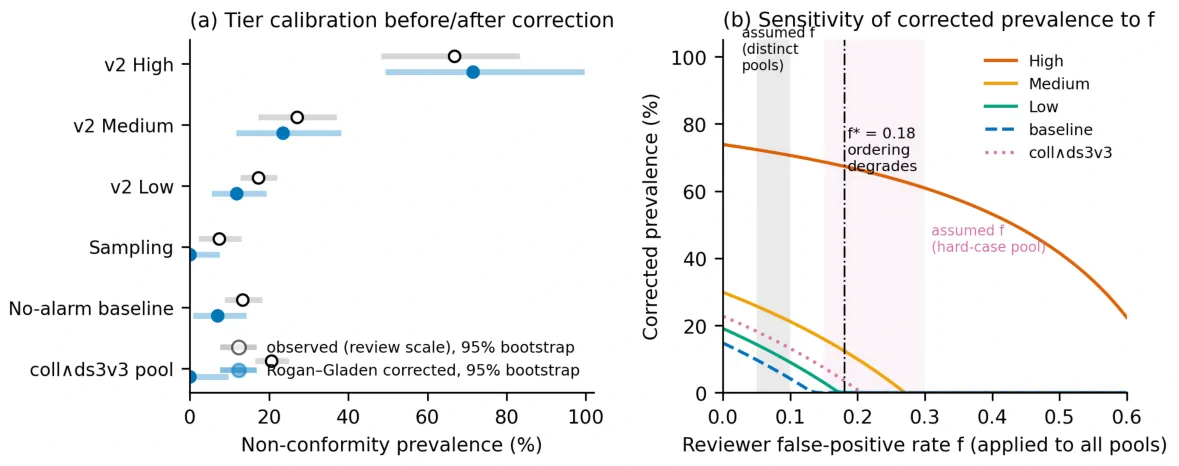
Label-noise correction and its sensitivity. (**a**) Non-conformity prevalence by v2 tier and key pools: observed review-scale estimates (open circles, grey bars = stratified-bootstrap 95% intervals) versus Rogan–Gladen-corrected estimates (filled circles, blue bars = bootstrap 95% intervals propagating review sampling error, the Jeffreys posterior of sensitivity *a*, and uniform draws of the pool-specific false-positive rate *f*). Correction steepens the tier contrast (high ≈ 72%, low ≈ 12%) and drives the collision∧ds3v3 pool toward zero. (**b**) Corrected prevalence as a function of a single *f* applied to all pools (the most adversarial simplification): the tier ordering high > medium > low > baseline is preserved for all f<0.18 (dash-dotted line); shaded windows mark the assumed *f* ranges for acoustically distinct pools (grey, 0.05–0.10) and the hard-case pool (pink, 0.15–0.30).

Pool-specific Rogan–Gladen correction (a=0.905; *f* intervals per Section 4.3) is summarized in Table 8 and Figure 5a.

Four consequences follow.

1. **Monotonicity Survives and Steepens.** Independent within-pool mislabeling compresses observed contrast; correction widens it (high 67→∼72%, low 17→∼11%). The v2 ordering is not a labeling artifact.
2. **The v1 “Low > Medium” Anomaly Dissolves.** The v1 low tier is dominated by impact-triggered pools whose review labels carry the highest false-positive rates; corrected, the collision∧ds3v3 pool falls from 21.0% observed to at most ∼8%, likely below the corrected medium tier. The apparent low-tier advantage was reviewer noise concentrated where listening is hardest.
3. **The v2 Decision to Demote Collision∧ds3v3 Agreement is Reinforced:** its corrected precision is plausibly ≤10%, i.e., worse than the no-alarm baseline before correction and comparable to it after.
4. **Missed-Detection Pessimism is Halved or Better.** The observed 13.3% control-stratum rate implied ∼14% true prevalence and ∼15% system capture. Corrected (4–10%), a substantial share of the 26 “missed faults” are plausibly review false positives on borderline recordings, consistent with the independent observation that an open-set outlier model is also blind to most of them (Section 6.3): recordings that neither the fused system nor an unrelated detector flags, and that were labeled non-normal in the hardest listening regime, are exactly where reviewer false positives concentrate.

### 5.6. Sensitivity Analysis

How much of Section 5.5 depends on the assumed *f* intervals? We answer with two instruments (Supplementary Code S13; B=10,000; seeds fixed). Stratified bootstrap.Resampling reviews within strata, drawing *a* from its Jeffreys posterior (Beta(19.5,2.5), from 19/21), and drawing *f* uniformly from each pool’s interval yields the intervals in Table 8 and three ordering probabilities:

- Corrected high > medium > low in 95.4% of replicates (82.9% under campaign-cluster resampling, which additionally resamples campaigns within each stratum and thereby prices between-campaign variation);
- Including the baseline (high > medium > low > baseline): 80.1% within-stratum, dropping to 62.7% under campaign-cluster resampling—we therefore state the baseline-inclusive chain as a directional finding rather than a probabilistic guarantee. Its weakest link is low versus baseline, whose corrected intervals overlap (6–19% versus 1–14%), consistent with the low tier’s designed role as a bounded sampling pool rather than a detector (Section 6.8);
- Corrected collision∧ds3v3 pool below the corrected medium tier in 99.9% (95.6% campaign-cluster)—the demotion decision is insensitive to every modeled uncertainty.

The campaign-cluster arm (Supplementary Table S6) resamples campaigns with replacement within each stratum and then review units within each drawn campaign, with design weights fixed; it asks whether the orderings survive between-campaign variation, not merely within-stratum sampling error. With nine campaigns the outer resampling is coarse and the cluster intervals are conservative; the tier-ordering and demotion conclusions survive it, while the baseline-inclusive chain does not at high probability, as stated above. The stratified bootstrap resamples three random quantities per replicate: (i) review labels, drawn nonparametrically within each stratum; (ii) reviewer sensitivity a∼Beta(19.5,2.5); and (iii) pool false-positive rate f∼U(poolinterval). Pseudocode and the full analysis script are provided in Supplementary Code S13.

***f*-sweep.** Applying a single *f* to all pools (the most adversarial simplification, since pool-specific difficulty is the mechanism at issue) and sweeping f∈[0,0.6]: the ordering high > medium > low > baseline holds for all f<0.18; beyond that the baseline and then the low tier truncate to zero and the ordering degrades to a weak ordering rather than reversing. No *f* in the swept range reverses any pairwise conclusion used in Section 5.5 or Section 6. Corrected-probability curves with the assumed windows shaded are shown in Figure 5b.

The one quantity that remains genuinely wide is the corrected high-tier probability (bootstrap 49–100%): with only 56 v2-high reviewed units and a hard-case-calibrated *a*, the data support “roughly two in three, plausibly more”, not a third significant digit. The next scheduled review batch (a census of the ∼320-unit v2 high tier per batch) is the designed remedy and is part of the deployed monitoring loop (Section 6.1).

## 6. Discussion

### 6.1. What Changed in Production

The v2 rule (Section 4.4) shipped as pure post-processing in the deployed detection package: grade and calibrated tier probability are appended to every unit record, while the v1 score *R* is retained as a reference column only. Runtime cost is zero (no new inference). Each tier prints its production share at the end of the run, turning the budget table into a drift monitor: if the high tier’s share drifts from 0.7% toward 1% or above, either the line or the detectors have moved and recalibration is triggered. The monitor is empirically grounded: the per-campaign high-tier share ranges from 0.24 to 1.85%, with campaigns 2DBF and 55C7 exceeding the 1% high-tier budget (Supplementary Table S4), so the share printout flags exactly the campaigns whose mix departs from the audited window.

### 6.2. Ablation: v1 Versus v2 on Identical Data

The audit data double as a like-for-like ablation of the two grading philosophies on identical units and identical labels. No detector, threshold, or datum was changed; only the grading logic differed (Table 9).

The comparison is conservative toward v1: both rules were evaluated on the review sample drawn under v1’s own stratification, reweighted to the population.

### 6.3. Budget-Constrained Enhancement: The Mel Leg

An open-set spectral outlier score (“mel”, an independently trained radial-manifold model reported in companion work [43]) was evaluated on the audited sample as a candidate fifth leg. As an alarm leg it fails the budget test: at the line-calibrated 1% FPR threshold it would add ≈2.6% alarm volume to recover only 2/26 (8%) of control-stratum faults. As a grading leg it is strong: within every tier, mel-triggered units are 1.4–2.1× more precise (low tier: 30.7% versus 14.8%), at zero added alarms. It was therefore admitted only as a low→medium promotion rule, with the threshold back-solved from the budget: promote until high + medium = 3.00% exactly (threshold 0.473; 119 units promoted; promoted-subgroup review precision 34% (14/41), above the medium tier’s 27.0% (weighted review scale)). An initially proposed looser threshold (0.193, the line’s 1% FPR point) was rejected because it would have pushed high+medium to 3.69%, violating the budget. This rejection is an instructive case of the operating point being set by capacity, not by ROC geometry. Because the mel threshold was back-solved on the audited data, we treat this enhancement as preliminary, budget-constrained evidence rather than validated performance; leave-one-campaign-out evaluation of the promoted subgroup gives 34.1% (14/41) with the deployed threshold and 27.8% (15/54) under per-fold refitting—above the low tier’s 15.9% and close to the medium tier’s 29.8% (unweighted)—supporting its role as a marginal, budget-bounded uplift (Supplementary Table S5).

### 6.4. Institutionalizing Reviewer Calibration

The blind-test instrument is cheap and reusable: a “question bank” of ∼20 normal plus ∼20 seeded-fault recordings per line, administered at reviewer onboarding and quarterly thereafter, keeps (a,b) current and makes every future review correctable. Seeded-fault rigs, which constitute physically manufactured ground truth, are the enabling asset; we recommend that each line maintain a small set.

### 6.5. Scope of the Calibration

The tier probabilities are properties of this nine-campaign mixture during this window, not of the rule. All nine campaigns come from a single production line, so this study carries no cross-line evidence of its own; the cross-line claim rests on the label-free calibration discipline reported in companion work [44]. Cross-line transfer requires re-calibration. The deployment package ships a five-step procedure: alarm-rate check, threshold re-fit on line normals, tier-share monitoring, periodic per-tier sampling of ∼50 units per batch, and a line-specific blind test. This procedure is an instance of the label-free calibration discipline whose bias analysis is given in companion work [44]. The three-tier share printout makes drift visible at zero cost.

### 6.6. Single Reviewer and Hard-Case-Only Specificity

All 1161 review labels and the blind test come from one experienced reviewer, who is the line’s only review resource. This is a structural constraint of the deployment rather than a design shortcut, so the calibration measures the deployed human–machine system, not an idealized annotator. Multi-reviewer arbitration is not available on this line and cannot be assumed in comparable installations; the seeded-fault blind test is precisely the instrument that makes a single-reviewer pipeline correctable, by measuring the one reviewer actually deployed (binary accuracy 68% on the hardest slice, sensitivity 0.905, specificity 0.421). The residual limitations are the blind test’s size (n=50) and coverage (one difficulty slice): *a* is tight enough for our use (misses 2/21 on the hardest cases), while *b* is measured only where listening is hardest and enters the analysis only as an interval bound plus sensitivity sweep. The natural next measurements, both feasible with the same single reviewer, are a second blind test on a weak-abnormal-noise slice (extending *b*’s coverage) and a blinded re-listen of the two highest-leverage small sets (the 26 control-stratum “missed faults” and the high-tier units without mel corroboration), which would bound intra-rater repeatability even without a second opinion. A formal intra-rater repeatability study (blinded re-listen after ≥2 weeks) is scheduled; pending it, the confidence-stratified accuracy (72% for high-confidence versus 25% for medium-confidence judgments) provides partial evidence that reviewer errors concentrate in identifiable low-confidence items.

### 6.7. Definition of the Positive Class

The positive class is actionable non-conformity, including invalid recordings (re-test required). Invalid recordings constitute 31/461 of the reviewed v1 high tier, and they concentrate in impact-triggered pools; lines wanting a pure fault probability should split the disposition (re-test versus repair) at review time. This is a definition choice, not a measurement limitation.

### 6.8. Low Tier as Designed Triage, Not Detection

Corrected low-tier prevalence (9–14%) sits near or above the corrected baseline (4–10%), an enrichment of roughly 1.3–2×. Its value is as a bounded sampling pool under the 9% budget, and the mel promotion shows how to mine it further; we state this plainly rather than claiming detection value that the data do not support. Two revision analyses sharpen this statement. Restricting the positive class to physical faults (invalid recordings excluded), the weighted low-tier rate (7.4%) falls below the fault-only no-alarm baseline (9.7%) while high and medium keep their order (64.8%/24.3%; Supplementary Table S9); invalid recordings—which concentrate in impact-triggered pools (51/55)—account for most of the low tier’s apparent enrichment. And under campaign-cluster resampling the baseline-inclusive ordering holds in only 62.7% of replicates (Section 5.6). Both point the same way: the low tier is a budgeted triage queue, and we claim no detection value for it.

### 6.9. Missed Detections as the System-Level Boundary

Even after correction, 4–10% of un-alarmed units are non-conforming, and the audit shows that the missed faults are “weak” signatures (audible to a careful listener, spectrally close to normal) that an unrelated open-set model also misses. Grading cannot fix this; more sensitive front-ends or acoustic features are the open problem. We flag it honestly as the residual risk that the budget system manages rather than eliminates.

### 6.10. Transferability

Nothing in the loop is compressor-specific: any fused detection system with human verification can (i) stratify review with a no-alarm control, (ii) measure its reviewers against physically seeded ground truth, (iii) correct pool-by-pool with Rogan–Gladen, and (iv) re-derive grading rules under explicit capacity budgets. The prerequisite asset, namely seeded-fault units—is standard equipment on many test lines already used for attribute gauge studies [31].

## 7. Conclusions

We converted a binary industrial alarm stream into calibrated, budget-constrained risk tiers by treating human review as a measurable instrument rather than as ground truth. A stratified audit refuted the multi-detector-agreement premise of the incumbent grading (high tier 25.2%, barely above the 13.3% no-alarm baseline) and localized the failure to correlated false positives (collision∧ds3v3 agreement: 21.0%, n=386). A seeded-fault blind test measured the reviewer (a=0.905, b=0.421 on hard cases), and pool-specific Rogan–Gladen correction then showed the corrected tier probabilities to be strictly monotone (≈72%/≈24%/≈12% against a 4–10% no-alarm baseline; ordering preserved in 95% of within-stratum and 83% of campaign-cluster bootstrap replicates, and under any common mislabel rate below 0.18), the low-tier anomaly to be a label-noise artifact, and the missed-detection rate to be roughly half its observed value. The corrected evidence was redeployed as an evidence-type grading rule meeting 1%/3%/9% review-capacity budgets, with an enhancement leg admitted strictly through the remaining budget. The audit–measure–correct–redeploy loop ran in days, not months, and each of its instruments—namely the control stratum, the seeded-fault question bank, the pooled prevalence correction, and the budget-first thresholds—is directly reusable in other human-verified detection systems.

## Figures and Tables

**Table 1:** Self-contained summary of the four detector legs (input, trigger threshold, and role). Full internal details, training data, and validation protocols remain in the companion manuscripts [42,43,44,45].

Leg	Input	Trigger Threshold	Role
collision	envelope/peak morphological features from the raw (un-normalized) 2.0 s record	P(impact)>0.468 (random-forest probability)	Impact faults
ds3v3	log-spectrogram of the 2.0 s record	CNN 4-class output, class > normal (normal/uncertain/suspect/alarm)	Broad-spectrum anomalies
phf	scalar periodic high-frequency noise strength (harmonic aggregation over the fault band [45])	phf>30	Periodic squeal/whine
burr	scalar high-frequency burr strength	burr>6.3 (offline re-check queue only)	Offline sampling only

**Table 2:** Capacity budgets and tier-to-disposition mapping specified by operations.

Tier	Budget (Share of Production)	Disposition
High	≤1%	Do not release; immediate off-line inspection/auscultation
Medium	≤3% cumulative	Same-shift review by line inspector
Low	≤9% cumulative	Trend logging; sampled checks as capacity permits
Sampling (burr-only)	—	Offline batch re-check queue

**Table 3:** Five-stratum review sample design. Stratum sizes were chosen to fit one review shift; the top tier is a full census.

Stratum	Design	*n*	Population
(i) v1 high	Census (every unit)	461	461
(ii) v1 medium	Stratified sample (by trigger composition)	200	919
(iii) v1 low	Stratified sample (by trigger leg × campaign)	200	2707
(iv) burr-only	Sample	100	485
(v) no-alarm control	Random sample of un-alarmed units	200	41,451

**Table 4:** Observed and HT-weighted review-scale precision by v1 tier (undecidables excluded). The intended ordering high > medium > low fails: the high tier barely exceeds the no-alarm baseline, and the low tier apparently outperforms the medium tier. Undecidable exclusions per stratum: 53/19/12/5/5, giving denominators 408/181/188/95/195. Within a stratum the design weights are constant, so the observed and HT-weighted stratum precisions coincide; the two differ for the v2 tiers (Table 5), which cut across strata.

v1 Tier	Observed	HT-Weighted	Wilson 95%
High (census, 461)	103/408 = 25.2%	25.2%	21–30%
Medium (200 sampled)	25/181 = 13.8%	13.8%	10–20%
Low (200 sampled)	49/188 = 26.1%	26.1%	20–33%
Burr-only (100)	7/95 = 7.4%	7.4%	4–14%
No-alarm control (200)	26/195 = 13.3%	13.3%	9–19%

**Table 6:** Observed review-scale precision by trigger pattern (strata (i)–(iii) pooled; undecidables excluded). Agreement patterns that include the periodicity feature phf reach 85.7% precision, whereas the collision∧ds3v3 agreement that dominated the v1 high tier achieves only 21.0%, below the single phf leg alone (40.0%).

Trigger Pattern	*n* Reviewed	Precision	Population
ds3v3∧phf	21	85.7%	23
collision∧ds3v3∧phf	7	42.9%	7
phf only	75	40.0%	1027
collision∧phf	13	30.8%	41
collision∧ds3v3	386	21.0%	480
collision only	152	20.4%	1822
ds3v3 only	116	8.6%	638
phf∧burr	6	0%	40

**Table 7:** Observed precision of phf-triggered units by phf strength (strata (i)–(iii); undecidables excluded). The ≥55 bin is a small-sample census (n=18); the design decision it supports rests on the whole ladder, not on the 100% point.

phf	Precision	Wilson 95%
[30,40)	22/79 = 27.8%	19–39%
[40,55)	15/25 = 60.0%	41–77%
≥55	18/18 = 100%	82–100%

**Table 8:** Pool-specific Rogan–Gladen correction. The “*f*-endpoints” column applies the estimator to the weighted point estimate at the two ends of the *f* interval (deterministic calculation); the bootstrap column additionally propagates review-sampling error and the posterior uncertainty of *a*. All values recomputed end-to-end from the raw review and blind-test files.

Pool	Observed (k/n→ Review Scale)	*f* Interval	Corrected, *f*-Endpoints	Corrected, Bootstrap 95%
v2 High	39/56 → 66.8%	0.05–0.10	71–72%	49–100%
v2 Medium	50/168 → 27.0%	0.05–0.10	21–26%	12–38%
v2 Low	88/553 → 17.2%	0.05–0.10	9–14%	6–19%
Sampling	7/95 → 7.4%	0.05–0.10	0–3%	0–8%
No-alarm control	26/195 → 13.3%	0.05–0.10	4–10%	1–14%
collision∧ds3v3 pool	81/386 → 20.6% (wt.)	0.15–0.30	0–7%	0–10%

**Table 9:** Like-for-like ablation of v1 and v2 grading on identical reviewed units and labels. Both rules are evaluated on the review sample drawn under v1’s own stratification, reweighted to the population; the comparison is therefore conservative toward v1.

	High	Medium	Low	Monotone?	Budgets Met?
v1 (score sum, quantile cuts)	25.2%	13.8%	26.1%	No	Rates only
v2 (evidence-type dispatch)	66.8%	27.0%	17.2%	Yes	Yes (0.70/2.74/8.88%)

## Data Availability

The anonymized review-outcome table (Table S1, 1161 records, with the mel leg score included), blind-test records (Table S2), and per-campaign audit table (Table S3) are provided as Supplementary Materials. The analysis code and all aggregated tables are provided as Tables S4–S6, Figure S7, Tables S8–S12 and Supplementary Code S13. Raw vibration waveforms are proprietary and cannot be shared publicly.

## Supplementary Materials

The following supporting information can be downloaded at: https://www.mdpi.com/article/10.3390/s26175564/s1, Table S1, anonymized review-outcome table (v2, 1161 records, including the mel leg score); Table S2, blind-test records (50 recordings); Table S3, per-campaign audit table (v1 high tier); Table S4, campaign totals (production totals and tier counts); Table S5, leave-one-campaign-out validation table; Table S6, campaign-cluster bootstrap table; Figure S7, per-pool *f*-sweep figure (with source data); Table S8, *f*-interval anchor table; Table S9, fault-only/invalid-only split table; Table S10, sensitivity/specificity confidence intervals and undecidable-mapping table; Table S11, sample-size and achieved-precision table; Table S12, anonymized model-family × campaign table; Code S13, Supplementary Code (v1/v2 scoring functions, thresholds, bootstrap and LOCO pseudocode, repro_from_S1.py, README).
