# Supplementary material for: From Alarms to Probabilities: Stratified Human Review, Label-Noise Correction, and Calibrated Risk Grading for Industrial Vibration Anomaly Detection
Source: Sensors (Basel). 2026 Sep 2;26(17):5564. doi: 10.3390/s26175564 (PMC13568055; doi:10.3390/s26175564)
Supplement: Supplementary file 1 [file sensors-26-05564-s001.zip › S0_Supplementary_List.pdf]

S1 S1_review_outcomes_v2.csv
Anonymized stratified-review outcome table (1,161 records), including the mel enhancement-leg score; identical to the submitted S1 plus one column (mel_radial).

S2 S2_blind_test.csv
Blind-test answer sheet and hardware ground-truth key (50 borderline recordings).

S3 S3_per_campaign_v1_high.csv
Per-campaign v1 high-tier audit table (reviewed, confirmed, precision, Wilson 95% CI).

S4 S4_campaign_totals.csv
Per-campaign production totals and tier counts (aggregate values only).

S5 S5_Table_S_loco.csv
Leave-one-campaign-out validation of the v2 rule (fixed-threshold vs per-fold refit), mel promoted-subgroup out-of-fold precision.

S6 S6_Table_S_cluster.csv
Campaign-cluster bootstrap ordering probabilities and corrected tier intervals.

S7 S7_Fig_S_fsweep.pdf
Per-pool independent f-sweeps with conclusion-flip critical values (source data: S7_fsweep_curves.csv).

S8 S8_Table_S_f_anchors.csv
Data anchors for pool-specific false-positive-rate intervals (p_obs, identifiability bound, blind-test upper bound, invalid-share bound, adopted interval).

S9 S9_Table_S_faultsplit.csv
Fault-only vs invalid-only precision by tier (positive-class split).

S10 S10_Table_S_ab_ci.csv
Reviewer sensitivity/specificity Wilson and Clopper-Pearson CIs, undecidable-mapping sensitivity, and b-posterior upper arm.

S11 S11_Table_S_samplesize.csv
Stratum sizes, achieved Wilson half-widths, and stratification keys.

S12 S12_Table_S_model_by_campaign.csv
Anonymized product-model-family (A/B) composition by campaign (two slices).

S13 S13_p3_supplementary_code_rev1.zip
Supplementary Code S13: v1/v2 grading formulas and thresholds, LOCO refit procedure, bootstrap pseudocode and fixed seeds; repro_from_S1.py reproduces Tables 4/7/8 from

the submitted CSVs using standard libraries only; README included.
