# Supplementary material for: From Alarms to Probabilities: Stratified Human Review, Label-Noise Correction, and Calibrated Risk Grading for Industrial Vibration Anomaly Detection
Source: Sensors (Basel). 2026 Sep 2;26(17):5564. doi: 10.3390/s26175564 (PMC13568055; doi:10.3390/s26175564)
Supplement: Supplementary file 1 [file sensors-26-05564-s001.zip › S7_Fig_S_fsweep.pdf]

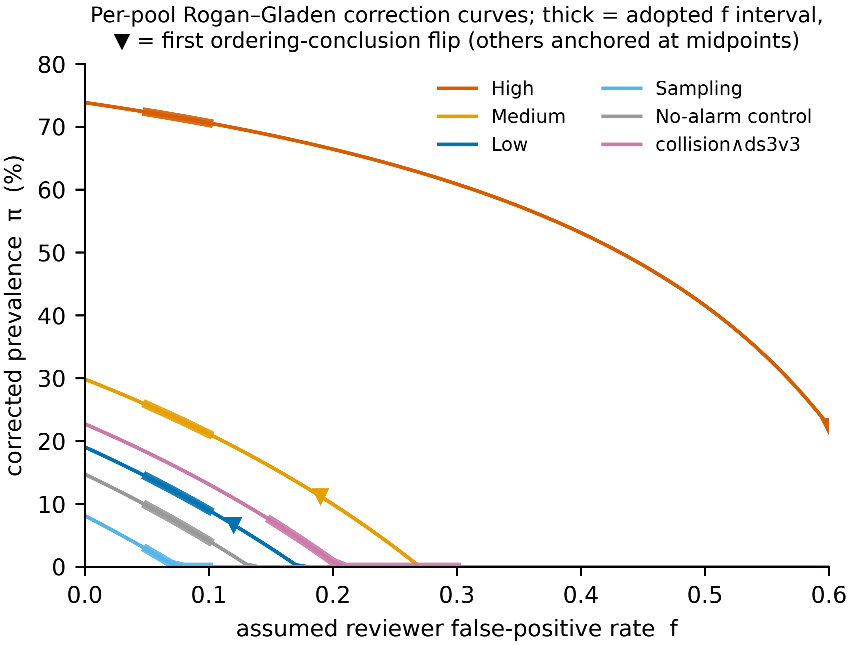
Per-pool Rogan–Gladen correction curves; thick = adopted f interval,
▼ = first ordering-conclusion flip (others anchored at midpoints)
High
Medium
Low
Sampling
No-alarm control
collision∧ds3v3
corrected prevalence π (%)
assumed reviewer false-positive rate f
0
10
20
30
40
50
60
70
80
0.0
0.1
0.2
0.3
0.4
0.5
0.6
